# Anti-inflammatory effects of apocynin on dextran sulfate sodium-induced mouse colitis model

**DOI:** 10.1371/journal.pone.0217642

**Published:** 2019-05-29

**Authors:** Young-Jae Hwang, Seung-Joo Nam, Wanjoo Chun, Song In Kim, Sung Chul Park, Chang Don Kang, Sung Joon Lee

**Affiliations:** 1 Department of Internal Medicine, Kangwon National University School of Medicine, Chuncheon, Korea; 2 Department of Pharmacology, Kangwon National University School of Medicine, Chuncheon, Korea; National Institutes of Health, UNITED STATES

## Abstract

**Background and aim:**

Various drugs have been developed for inflammatory bowel disease (IBD), but still there are limitations in the treatment due to the insufficient responses and significant adverse effects of immunosuppressant. Apocynin is an NADPH-oxidase inhibitor with established safety profiles. We aimed to investigate the protective efficacy of apocynin in IBD using chemical-induced mouse colitis model.

**Method:**

We induced experimental colitis by administrating 5% dextran sulfate sodium (DSS) to 8-week old BALB/c mouse for 11 days. Apocynin (400 mg/kg) or sulfasalazine (150 mg/kg) were administeredduring7 days. We monitored bodyweight daily and harvested colon and spleen at day 11 to check weight and length. We also examined histopathologic change and pro-, anti-inflammatory cytokines and enzymes from harvested colons (iNOS, COX-2, TNF-α, MCP-1, p-NrF2, and HO-1).

**Result:**

Apocynin significantly alleviated weight reduction induced by DSS treatment (21.64 ± 0.55 for Apocynin group vs. 20.33 ± 0.90 for DSS group, *p* = 0.005). Anti-inflammatory efficacy of apocynin was also shown by the recovery of colon weight and length. Histopathologic examination revealed significantly reduced inflammatory foci and erosions by apocynin treatment. Colonic expression of iNOS, COX-2, TNF-α, and MCP-1 was decreased significantly in the apocynin treated group. Anti-inflammatory mediators Nrf2 and HO-1 were activated significantly in apocynin treated mouse.

**Conclusion:**

Apocynin showed significant anti-inflammatory efficacy against chemically induced colonic inflammation. This study also revealed the unique action of apocynin compared to the currently prescribed drug, sulfasalazine. Given its excellent safety profile and potent efficacy with novel action mechanism, apocynin can be a new therapeutic molecule for the IBD treatment, which can be added to the currently available drugs.

## Introduction

Inflammatory bowel disease (IBD) is chronic progressive inflammatory disease associated with infiltration of leukocytes, crypt destruction, edema, and ulcerations. The incidence and prevalence of IBD have been increasing worldwide [1]. Steroids, azathioprine, and several biologic agents such as infliximab and adalimumab are the most commonly used drugs for IBD patients. But these agents have serious side effects such as infection and malignancy [2]. Lots of new agents have been developed showing promising efficacy, but many of these agents have been withdrawn from the market due to their adverse effects [3,4]. Still, many new drugs are being actively investigated for more safe and effective treatment [5].

Apocynin (4-hydroxy-3-methoxyacetophenone) is phytochemical extracted from the root of the medicinal herb *Picrorhizakurroa* [6]. Apocynin is the well-known inhibitor of nicotinamide adenine dinucleotide phosphate (NADPH)-oxidase, and its action mechanism is known to inhibit assembly of functional NADPH-oxidase complex [7]. NADPH-oxidase, located in the plasma membrane of phagocytes such as macrophages and polymorphonuclear leukocytes, is known to be associated with various inflammatory diseases including atherosclerosis, asthma, arthritis, and Crohn’ disease [7–11]. Several studies have shown the anti-inflammatory efficacy of apocynin on these chronic inflammatory diseases [7,12]. Aberration in NADPH-oxidase is one of the important pathologies of IBD [11,13]. So apocynin can be a new candidate molecule for the treatment of IBD in consideration of its well-known anti-inflammatory effect and excellent safety profile.

From this background, we aimed to investigate the protective effect of apocynin on IBD and discover molecules associated with its action mechanism using a chemical-induced mouse colitis model.

## Method and material

### Animals

Male BALB/c mice (age, eight weeks; weight, 20~25g) were purchased from Dae-Han Biolink (Seoul, Korea). Every mouse was housed in a temperature-controlled room (at 23°C) with a 12/12-hour light/dark cycle under specific pathogen-free conditions, in polystyrene cages at Kangwon National University. Mice were euthanized for sample collection and histologic examination at the end of the study by CO_2_ chamber. The procedures were in accordance with the ARRIVE (Animals in Research: Reporting In Vivo Experiments) statement. Ethical approval for the present study was provided by the Committee Animal Care and Use Committee at the Kangwon National University (KIA-CUC-11-0012).

### Dextran sulfate sodium-induced colitis model

The mice were divided into four experimental groups (each group, n = 6): healthy control group (Control group), colitis induced by dextran sulfate sodium group (DSS group), DSS with apocynin group (Apocynin group), and DSS with sulfasalazine group (Sulfasalazine group). Control group was defined as the negative control and DSS group defined as the positive control in this study. The mice of the control group received tap water with no additives. Except for the control group, all other groups were given drinking water supplemented with 5% DSS. Water (Control and DSS group), apocynin (400 mg/kg) and sulfasalazine (150 mg/kg) were administrated by oral route using sonde during seven days. Apocynin was purchased from Cayman Chemical (Ann Arbor, MI) and dissolved in dimethyl sulfoxide.

### Mouse body and organ measurement

We checked mice daily during the experiment period whether there were signs of mice distress like chilling, poor grooming, and declined activity. We measured the weight of each mouse every day (Day 1–11). Mice were euthanized by CO_2_ asphyxiation at the end of the experiment on day 11. We sprayed 70% ethanol onto the mice body and carefully opened the mice abdomen by ventral midline incision. We harvested spleens and colons (from the cecum to rectum) and measured colon length, colon weight, and spleen weight as described previously [14].

### Macroscopic feature and histological examination of mice colon

We opened extracted colons longitudinally and washed out the stools with phosphate buffered saline. The whole colonic segments were fixed with phosphate-buffered formalin and were stained with hematoxylin and eosin. We analyzed the state of crypt structure and infiltration of inflammatory cells for the whole colon. We scored the degree of colonic inflammation as described before with slight modification [15]. To reduce sampling bias, we evaluated the inflammatory cell infiltration and intestinal architectural change for the whole colon. Inflammatory cell infiltration was evaluated by counting the number of areas with large lymphoid aggregates occupying more than half of the lamina propria and scored as follows: 0 for no significant lymphoid aggregates, 1 for 1–3 areas, 2 for 4–6 areas, and 3 for more than 7 areas of large lymphoid aggregates in the whole colon. Intestinal architectural change was assessed by scoring the degree of erosions and ulcerations as follows: 1 for focal erosions, 2 for erosions ± focal ulcerations, and 3 for extensive ulcerations in the whole colon. Total histological score represents the sum of the infiltration and architectural change scores.

### Western blot analysis

Western blot analysis was performed for analyzing pro- and anti-inflammatory enzymes and cytokines including inducible nitric oxide synthase (iNOS), cyclooxygenase-2 (COX-2), monocyte chemoattractant protein-1 (MCP-1), tumor necrosis factor-α (TNF-α), nuclear factor E2-related factor-2 (Nrf2), phosphorylated-nuclear factor E2-related factor-2 (p-Nrf2), and heme oxygenase-1 (HO-1). Harvested colons were lysed in PRO-PREP protein extraction solution (iNtRONBiotechnology, Korea), and sonicated on ice. Protein concentrations in the supernatants were determined using the bicinchoninic acid assay (Sigma). Equal amounts of proteins were diluted with 2X protein loading buffer (0.25 M Tris-HCl, pH 6.8, 5 mM EDTA, 5 mM EGTA, 25 mM dithiothreitol, 2% SDS, and 10% glycerol with bromophenol blue as the tracking dye), incubated in a boiling water bath for 5 minutes, separated on 8~15% SDS-polyacrylamide gels or gradient gels, transferred to PVDF membrane (GE Healthcare), and blots were blocked in 5% nonfat dry milk in TBST (20 mM Tris-HCl, pH 7.6, 137 mM NaCl, 0.05% Tween 20) for 1 hour at room temperature, and probed with indicated antibodies in the same buffer overnight at 4°C. The membranes were then washed with TBST and incubated with HRP-conjugated goat anti-rabbit IgG (Jackson ImmunoResearch) or HRP-conjugated goat anti-mouse IgG (Jackson ImmunoResearch) for 2 hours at room temperature. The membranes were rinsed with TBST. The blots were visualized with enhanced chemiluminescence (GE Healthcare) and exposed on film (Kodak). Gel images were scanned and quantified using Image Quant software (Molecular Dynamics, Sunnyvale, California, USA). For a fair comparison, β-actin was used as a loading control. The following antibodies were used in the present study; iNOS (BD Biosciences), COX-2, Nrf2 (Santa Cruz Biotechnology Inc), MCP-1, TNF-α, p-Nrf2, HO-1 (Abcam), and β-actin (Sigma). The results of pro-inflammatory enzymes and cytokines from colon cells were expressed as fold changes of control.

### Statistical analyses

All values shown in the figures are expressed as the mean ± SD obtained from at least three independent experiments. Statistical analysis was carried out by one-way analysis of variance (ANOVA) with Tukey’s post hoc test, and Wilcoxon signed ranks test using SPSS software 19K (SPSS, Chicago, IL, USA). * (*p*<0.05) and ** (*p*<0.01) were indicated statistically significant difference compared to DSS group.

## Result

### Measurement of mouse body weight and organ change

**Fig 1:**
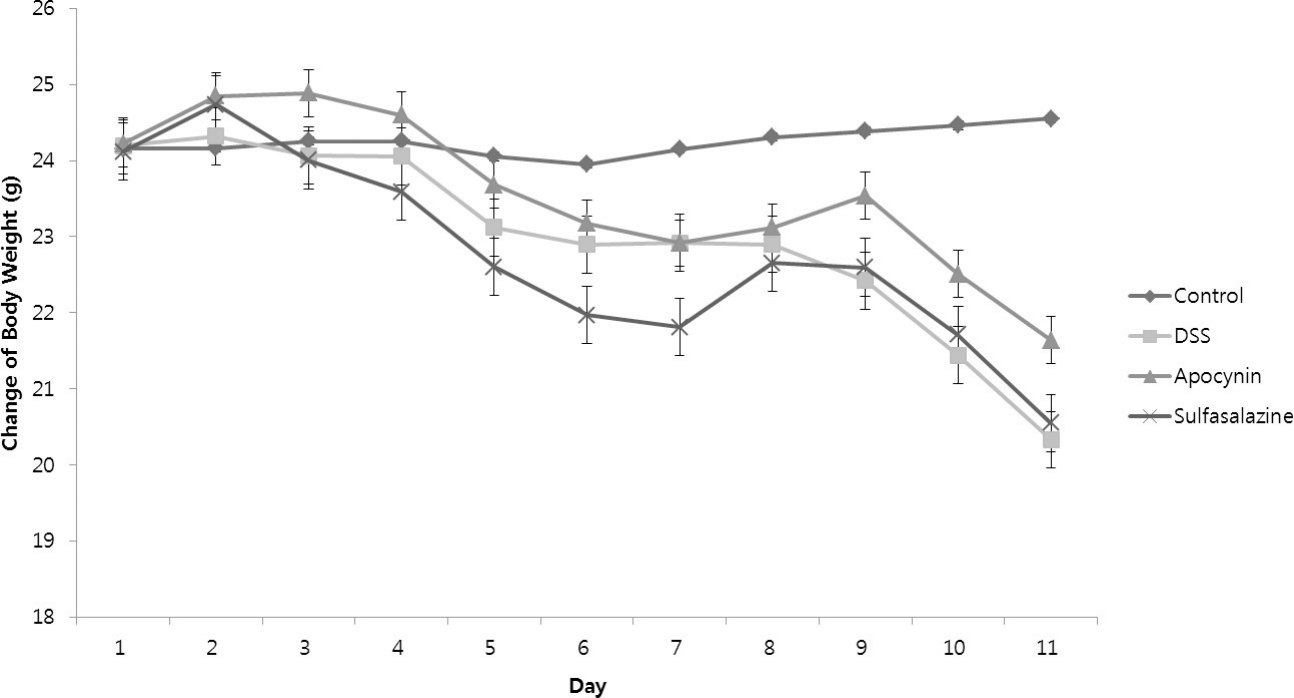
Serial change of mouse body weight in each group. Mouse body weight of Control, DSS, Apocynin and Sulfasalazine group during the study period (n = 6, bars represents means ± SD).

**Fig 2:**
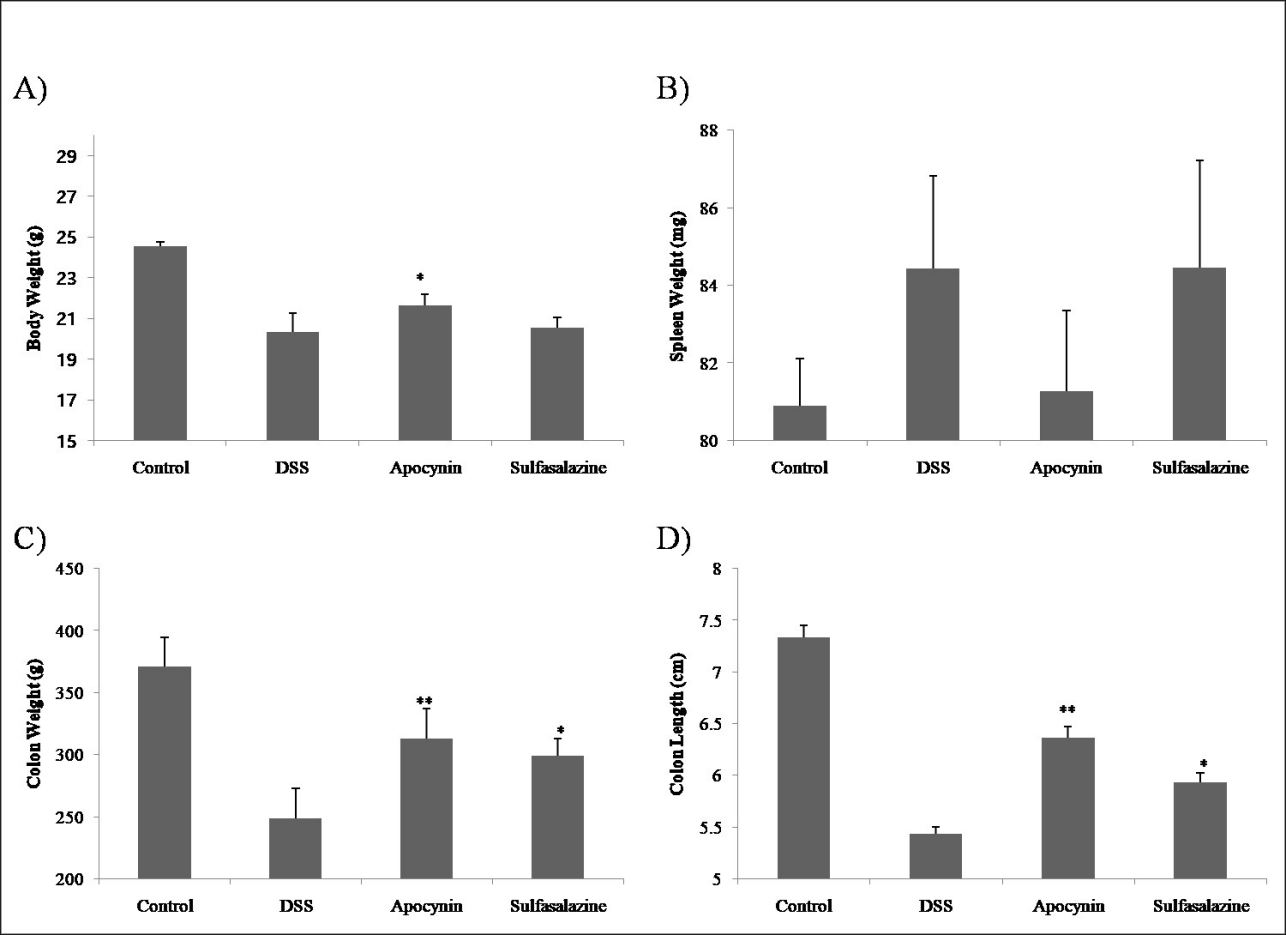
Difference of body weight, spleen weight, colon weight and colon length between each group. Effect of apocynin on the body weight (A), spleen weight (B), colon weight (C), and colon length (D) in Control, DSS, Apocynin and Sulfasalazine group at day 11 (n = 6). *(*p* < 0.05) and **(*p* < 0.01) indicate statistically significant difference compared to treatment with DSS alone.

We identified the protective effect of apocynin by measuring changes in body weight, spleen weight, colon length, and colon weight (Figs 1 and 2, Table 1). There was no interval change in body weight of the control group (Day 1, 24.15±0.55g vs. Day 11, 24.55±0.22g, *p* = 0.173) (Fig 1). Except for thecontrol group, all of the other groups showed a significant reduction in body weight between pre-treatment (Day 1) and post-treatment (Day 11). For DSS only group, there was a continuous reduction in body weight (Fig 1). But in Apocynin and Sulfasalazine group, recovery of body weight was observed at Day 8 and 9. At Day 11, body weight was significantly different between apocynin group and DSS group (21.64±0.55g vs. 20.33±0.90, *p* = 0.005) (Fig 2A, Table 1). Also, weight loss in Apocynin group was less than Sulfasalazine group (21.64±0.55g vs. 20.55±0.48g, *p* = 0.023; CI: 0.13 to 2.04).

**Table 1:** Assessment of macroscopic characteristics at day 11 for Control, DSS, Apocynin and Sulfasalazine group.

Group	Control	DSS (ref)	DSS with apocynin	DSS with sulfasalazine
**Body weight (g)**	24.55±0.22^a^	20.33±0.90	21.64±0.55^a^	20.55±0.48
**Colon weight (mg)**	371.12±23.34^a^	248.55±24.07	313.18±23.87^a^	299.37±13.54^a^
**Colon length (cm)**	7.33±0.29^a^	5.43±0.18	6.37±0.27^a^	5.93±0.23^a^
**Colon weight/length ratio**	50.57±1.36	45.76±4.33	49.17±2.52	50.56±3.98
**Spleen weight (mg)**	80.90±3.00	84.43±5.87	81.27±5.09	84.45±6.80

Data are presented as mean ± SD (n = 6)

DSS, Dextran sulfate sodium

^a^Statistical significance (*p*< 0.01) compared to DSS group.

Colon weight was significantly reduced for DSS treated mice, but in Apocynin group, colon weight reduction was lesser (313.18 ± 23.87 mg vs. 248.55 ± 24.07 mg, *p* < 0.001) (Fig 2C, Table 1). Colon weight reduction of Sulfasalazine group was also lesser than DSS group (299.37 ± 13.54 mg vs. 248.55 ± 24.07 mg, *p* = 0.001). There was no significant difference in colon weight between Apocynin group and Sulfasalazine group (313.18 ± 23.87 mg vs. 299.37 ± 13.54 mg, *p* = 0.688). DSS induced colitis resulted in significant colon length reduction (Fig 2D, Table 1). Apocynin and sulfasalazine treatment ameliorated colon length reduction significantly. Colon weight/length ratio showed recovery of this value in Apocynin and Sulfasalazine group compared to DSS group, even though this was not statistically significant (Table 1). Spleen weight showed no significant difference among treatment groups, but DSS treatment tended to increase spleen weight and apocynin treatment alleviate weight increase (Fig 2B, Tables 1 and 2).

**Table 2:** Comparison of spleen weight at day 11 for control, DSS, apocynin and sulfasalazine group.

Group	Spleen weight (mg)	*p*-value (ref: control)	*p*-value (ref: DSS)
**Control**	80.90±3.00		
**DSS**	84.43±5.87	0.671	
**DSS with apocynin**	81.27±5.09	0.999	0.740
**DSS with sulfasalazine**	84.45±6.80	0.668	1.000

Data are presented as mean ± SD (n = 6)

DSS, Dextran sulfate sodium

### Histopathologic examination of colon

**Fig 3:**
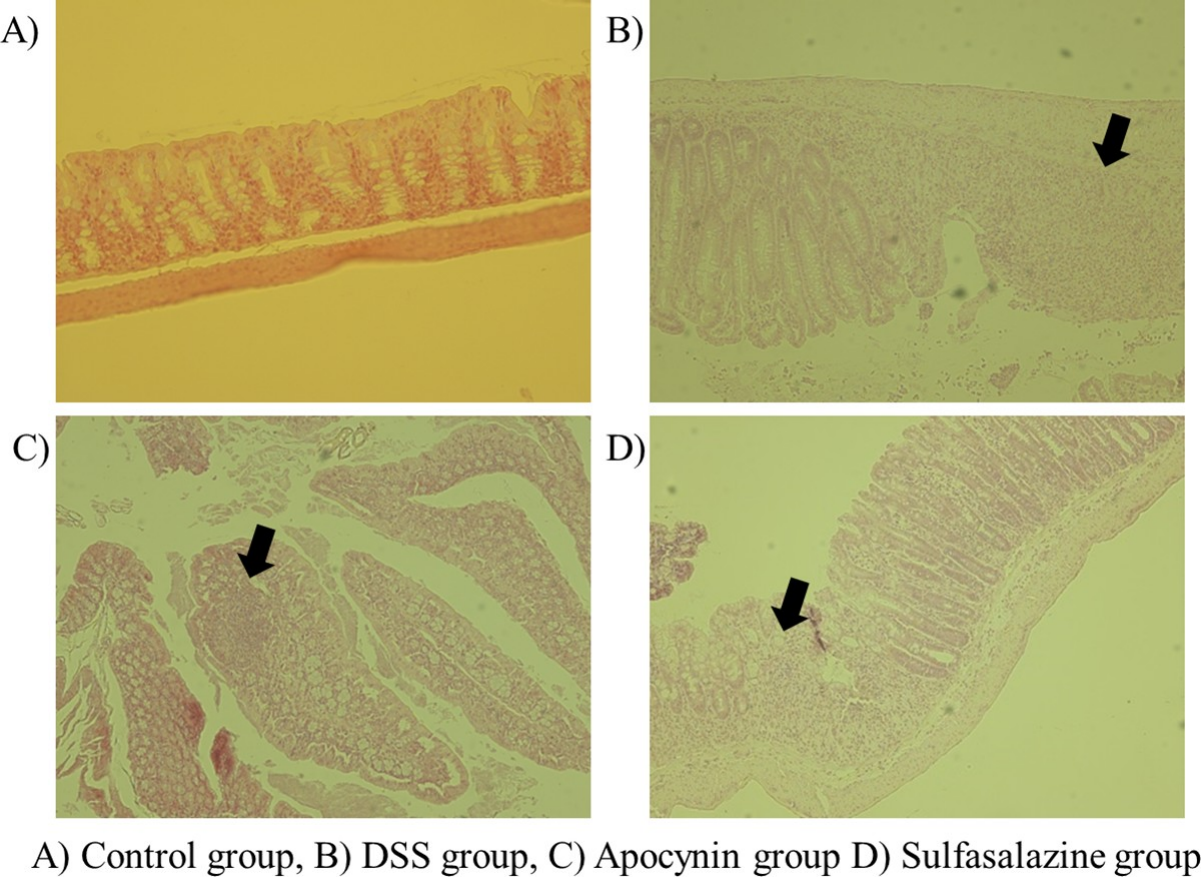
Histopathologic comparison between each group. Histopathologic examination of mouse colon harvested at day 11 of Control (A), DSS (B), Apocynin (C) and Sulfasalazine group (D). Arrows indicate inflammatory foci (H&E stained, magnification 100X).

Microscopic examination of colon specimens from the Control group showed normal epithelium with well-defined crypt length and no infiltration of inflammatory cells in colonic mucosa (Fig 3A). Colon specimen from DSS treated mice showed destructed epithelial integrity with shortening of crypts, erosions, and infiltration of inflammatory cells (Fig 3B). Compared with DSS group, colon specimens with apocynin and sulfasalazine treated mice showed less affected inflammatory change by DSS. The crypt was relatively intact, and epithelial loss was not severe with mild infiltration of inflammatory cells (Fig 3C and 3D). Histopathologic scores also increased significantly after the treatment of DSS, but apocynin significantly ameliorated the intestinal damages, especially for the inflammatory cell infiltrates into the colonic mucosa (Table 3).

**Table 3:** Histopathologic scores of the colonic inflammation.

Group	Control	DSS	Apocynin	Sulfasalazine
**Lymphoid aggregates**	0	2.60 ± 0.548	0.80 ± 0.837	2.00 ± 0.707
***→p*-value**^**a**^	na	<0.001	0.206	<0.001
***→p*-value**^**b**^	<0.001	na	0.001	0.433
**Erosion/ulceration**	0	2.80 ± 0.45	2.00 ± 0.71	2.20 ± 0.45
***→p*-value**^**a**^	na	<0.001	<0.001	<0.001
***→p*-value**^**b**^	<0.001	Na	0.072	0.229
**Total score**	0	5.40 ± 0.548	2.80 ± 1.304	4.20 ± 0.837
***→p*-value**^**c**^	na	<0.001	0.051	0.002
***→p*-value**^**d**^	<0.001	na	0.047	0.176

Data are presented as mean ± SD (n = 5)

DSS, Dextran sulfate sodium; na, not applicable

^a^*p*-value calculated using the Tukey's honest significance test with the control group as reference

^b^*p*-value calculated using the Tukey's honest significance test with the DSS group as reference

^c^*p*-value calculated using the Tamhane’s T2 test with the control group as reference

^d^*p*-value calculated using the Tamhane’s T2 test with the DSS group as reference

### Effect of apocynin on pro-inflammatory enzymes and cytokines in the colon

**Fig 4:**
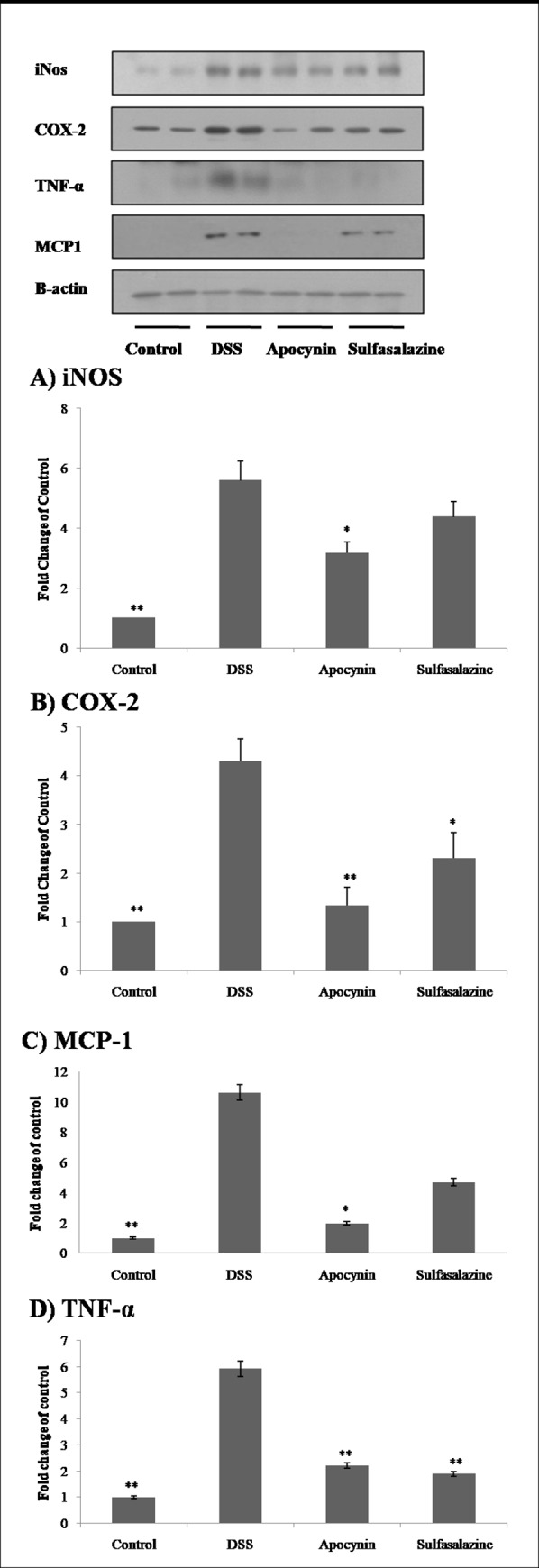
Comparison of pro-inflammatory mediators between each group. Multiple protein bands showed protein expression of pro-inflammatory mediators (iNOS, COX-2, TNF-α, and MCP-1) in mouse colon of Control, DSS, Apocynin and Sulfasalazine group by Western blot analysis. The graphs showed comparison of fold change in each group. The data were obtained from three mice for each group and expressed as fold change of control. Apocynin group showed significantly reduced expression of pro-inflammatory mediators (iNOS (A), COX-2 (B), MCP-1 (C) and TNF-α (D)). * (*p* < 0.05) and ** (*p* < 0.01) indicate statistically significant differences from treatment with DSS alone.

Overexpression of iNOS and COX-2 in the colon has an important role in the inflammatory pathway of IBD [16]. We examined the effect of apocynin on these pro-inflammatory enzymes. The protein levels of iNOS and COX-2 were determined by Western blot analysis. The expression of iNOS was increased in the colon specimens of DSS treated mice. Apocynin treatment significantly reduced iNOS expression (*p* = 0.022, Fig 4A). Also, COX-2 expressions induced by DSS were significantly reduced in Apocynin and Sulfasalazine groups (Apocynin group: *p* = 0.003; Sulfasalazine group: *p* = 0.031, Fig 4B).

We also examined the effect of apocynin on the pro-inflammatory cytokines MCP-1 and TNF-α by Western blot analysis. MCP-1 initially identified as a chemotactic cytokine with potent monocyte attracting properties acts on various immune cells including T cells and NK cells and is known to be elevated in IBD patients [17–19]. The expression of MCP-1 was also significantly reduced in Apocynin group compared with DSS group (*p* = 0.011, Fig 4C). Sulfasalazine group did not show the statistically significant reduction in the MCP-1 expression (*p* = 0.070). TNF-α production induced by DSS treatment was also significantly reduced in Apocynin and Sulfasalazine group compared with DSS group (*p <*0.001 and *p <*0.001, respectively; Fig 4D).

### Effect of apocynin in the anti-inflammatory pathway of colon

**Fig 5:**
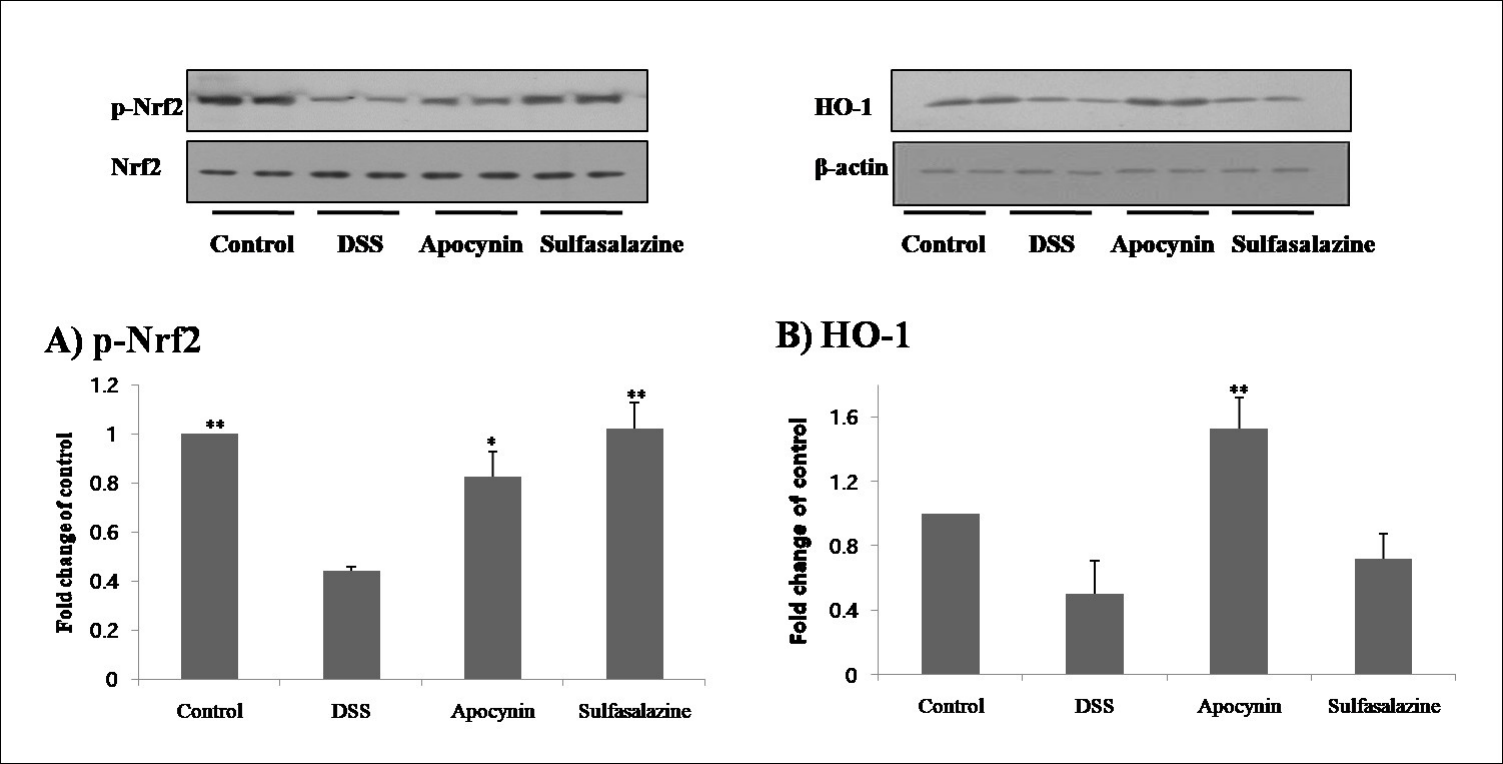
Comparison of anti-inflammatory enzymes between each group. Activation of anti-inflammatory enzymes (p-Nrf2 and HO-1) after apocynin treatment on DSS-induced colitis model. The data were obtained from three mice for each group and expressed as fold change of control. * (*p* < 0.05) and ** (*p* < 0.01) indicate statistically significant differences from treatment with DSS alone.

We examined the molecules involved in the anti-inflammatory pathway by analyzing Nrf2 and HO-1. p-Nrf2, the activated form of Nrf2, was oxidative stress marker and regulates a broad spectrum of oxidative stress protective genes [20,21].HO-1 is a stress-responsive protein [22]. Both Nrf2 and HO-1 are considered as potential therapeutic targets for IBD [20–22]. In our study, apocynin significantly induced activation of Nrf2 (*p* = 0.024) and this action was also observed in sulfasalazine (*p* = 0.002) (Fig 5). Interestingly, HO-1 production was induced only by apocynin treatment (*p* = 0.009), and sulfasalazine did not induce significant HO-1 production (*p* = 0.779) (Fig 5).

## Discussion

Apocynin is a well-known NADPH-oxidase inhibitor, and there have been several studies on the therapeutic effect of apocynin in chronic inflammatory diseases such as atherosclerosis [7], rheumatoid arthritis [10], airway inflammation [9], stroke and IBD [12,13]. Many in vitro and in vivo experiments have shown the therapeutic potential of apocynin as an anti-inflammatory agent. Also, there are phase I clinical trials showing promising efficacy and safety of apocynin [23,24]. However, the exact action mechanism of apocynin has not been fully elucidated yet.

UC develops through various inflammatory pathway [25,26]. Disruption of the epithelial barrier causes increased permeability of the intestinal epithelium, resulting in increased uptake of luminal antigens. Macrophages and dendritic cells recognize non-pathogenic microbiota through the molecular pattern-recognition receptors and are changed to activated phenotype. Activation of NF-kB pathway stimulates the transcription of various genes resulting in the production of pro-inflammatory cytokines (TNF-α, IL-1β, and IL-6) [12,26]. NADPH-oxidase stimulated by TNF-α, IL-1β, and IL-6 catalyzes the production of a superoxide free radical [13,27–29]. Oxidative stress in the intestine was considered an important factor of pathogenesis and progression of IBD [13]. ROS promotes apoptosis and necrosis by inducing endothelial dysfunction and oxidative damage of DNA, protein, and lipid [13,21]. NOS and COXs are also participated in ROS generation by catalyzing chemical reactions [21].

In this study, using an experimental DSS-induced colitis mouse model, we examined the role of apocynin on intestinal epithelial cells with inflammation. Protective effect of apocynin was evident by colon weight and length. We also showed that colonic expression of iNOS, COX2, TNF-α, and MCP-1 induced by DSS was significantly attenuated by the apocynin treatment revealing the protective effect of apocynin in IBD. The roles of these pro-inflammatory enzymes and cytokines are well known in the human colonic epithelium of UC [25]. We also identified that anti-inflammatory mediators of Nrf2 and HO-1 were significantly activated by apocynin treatment. Several studies have shown significant roles of antioxidant therapy in the management of IBD [21,30]. Exposed to oxidative stress, Nrf2 was known to suppress protein kinase C and decrease NADPH-oxidase activation and ROS production [31,32]. In IBD, activation of HO-1 has a defensive mechanism to reduce inflammation and tissue damage in the intestinal mucosa, so HO-1 has received attention as a new therapeutic target of IBD recently [22,33]. From our study, we can expect that apocynin will have a significant therapeutic effect by reducing pro-inflammatory cytokines and inducing anti-inflammatory molecules of Nrf2 and HO-1.

**Fig 6:**
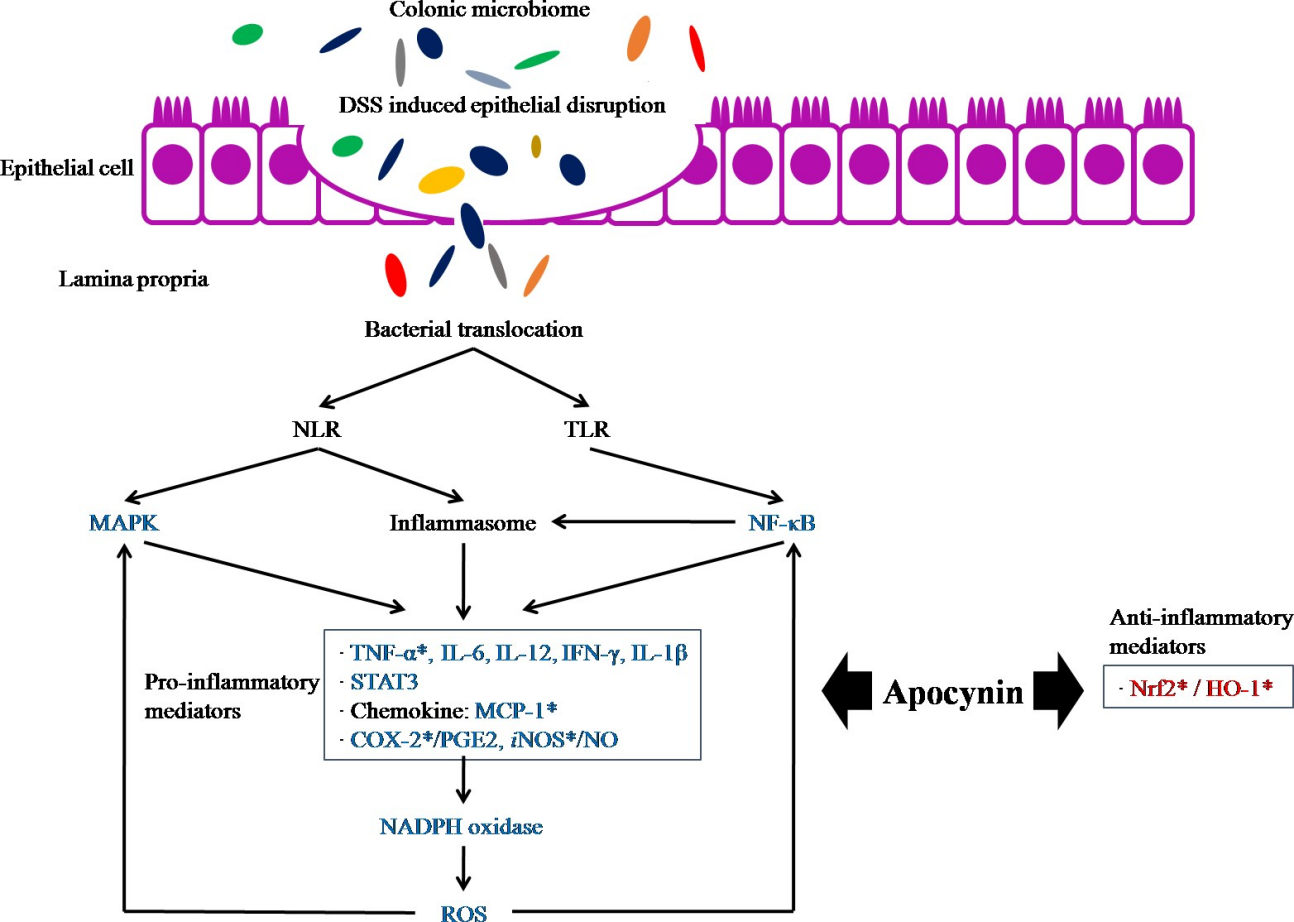
Proposed pathologic mechanisms of DSS-induced colitis and summary of the published efficacy of apocynin on the inflammatory mediators of IBD mouse model. Blue letters indicate studied mediators which are inhibited by apocynin, and red letters indicate mediators activated by apocynin.The asterisks indicate molecules analyzed in this study.NLR: Nod-like receptors, TLR: Toll-like receptors, MAPK: mitogen-activated protein kinases, ROS: reactive oxygen species, NF-κB: nuclear factor kappa B, STAT3: Signal transducer and activator of transcription 3,PGE2: prostaglandin E2, MCP-1:Monocyte chemoattractant protein 1, iNOS: inducible nitric oxide synthase, Nrf2:nuclear factor erythroid-derived 2-related factor 2, HO-1: heme oxygenase-1.

There have been many studies identifying the anti-inflammatory effect of apocynin in various diseases status, but there are limited studies on the protective effect of apocynin focusing on inflammatory bowel disease. Marin M et al. reported a protective effect of apocynin in an experimental murine colitis model induced by DSS [12]. They showed that NO and PGE_2_ production, as well as the iNOS and COX-2 expression, was reduced in apocynin treated colitis group. NF-κB and STAT3 were significantly downregulated in apocynin treated group. They also compared apocynin with sulfasalazine which is a widely used drug in clinics, but could not reveal a significant difference. Mouzaoui S et al. investigated the preventive effect of apocynin using the mouse model of acute colitis induced by TNF-α [27]. They showed that TNF-α induces colitis through ROS production by NADPH-oxidase, and these inflammatory pathways can be inhibited by apocynin. Ramonaite R et al. showed protective action of apocynin in an in vitro setting using DSS induced colitis mouse model [11]. In this study, apocynin increased cell viability and decreased the levels of TNF-α in the LPS-treated colonic epithelial cells isolated from DSS treated mouse. The above mentioned actions of apocynin on the various colonic inflammatory mediators in IBD mouse model are summarized in Fig 6.

Compared to previous studies, our study has several new findings. We evaluated chemokine MCP-1 which plays a critical role in colitis and elevated in IBD patients and found that apocynin treatment significantly reduce MCP-1 expression [17–19]. Interestingly, this effect was not significant by sulfasalazine treatment. HO-1 was also induced significantly by apocynin treatment, but not by sulfasalazine treatment. These findings show the possibility of apocynin as an additional therapeutic option being used synergistically with current sulfasalazine or 5-aminosalicylic acid in the treatment of ulcerative colitis.

## Conclusion

The inhibition of NADPH-oxidase in apocynin represents an attractive therapeutic strategy for various chronic diseases. Apocynin reduces the level of proinflammatory cytokines iNOS, COX-2, TNF-α, and MCP-1, which are important mediators of IBD. Also, apocynin activated anti‐inflammatory pathway by inducing activation of p-Nrf2 and production of HO‐1. Apocynin has different anti-inflammatory mechanism compared to currently used sulfasalazine in respect of MCP-1 and HO-1. This result shows the possibility of apocynin as a new therapeutic molecule which can be combined with currently used drugs.

## Abbreviations

IBD: inflammatory bowel disease
NADPH: nicotinamide adenine dinucleotide phosphate
DSS: dextran sulfate sodium
iNOS: inducible nitric oxide synthase
COX-2: cyclooxygenase-2
MCP-1: monocyte chemoattractant protein-1
TNF- α: tumor necrosis factor-α
Nrf2: nuclear factor E2-related factor-2
p-Nrf2: phosphorylated-nuclear factor E2-related factor-2
HO-1: heme oxygenase-1
